# Psychotherapy integration under scrutiny: investigating the impact of integrating emotion-focused components into a CBT-based approach: a study protocol of a randomized controlled trial

**DOI:** 10.1186/s12888-016-1136-7

**Published:** 2016-11-24

**Authors:** Anna Babl, Martin grosse Holtforth, Sara Heer, Mu Lin, Annabarbara Stähli, Dominique Holstein, Martina Belz, Yvonne Egenolf, Eveline Frischknecht, Fabian Ramseyer, Daniel Regli, Emma Schmied, Christoph Flückiger, Jeannette Brodbeck, Thomas Berger, Franz Caspar

**Affiliations:** Department of Clinical Psychology and Psychotherapy, University of Bern, Fabrikstrasse 8, 3012 Bern, Switzerland

**Keywords:** Emotion-Focused Therapy, Integration, Self-regulation, Psychological Therapy, Cognitive-behavioral therapy, Randomized Controlled Trial

## Abstract

**Background:**

This currently recruiting randomized controlled trial investigates the effects of integrating components of Emotion-Focused Therapy (EFT) into Psychological Therapy (PT), an integrative form of cognitive-behavioral therapy in a manner that is directly mirroring common integrative practice in the sense of assimilative integration. Aims of the study are to understand how both, an existing therapy approach as well as the elements to be integrated, are affected by the integration and to clarify the role of emotional processing as a mediator of therapy outcome.

**Methods:**

A total of 130 adults with a diagnosed unipolar depressive, anxiety or adjustment disorder (seeking treatment at a psychotherapy outpatient clinic) are randomized to either treatment as usual (PT) with integrated emotion-focused components (TAU + EFT) or PT (TAU). Primary outcome variables are psychopathology and symptom severity at the end of therapy and at follow up; secondary outcome variables are interpersonal problems, psychological wellbeing, quality of life, attainment of individual therapy goals, and emotional competency. Furthermore, process variables such as the quality of the therapeutic relationship are studied as well as aptitude-treatment interactions. Variables are assessed at baseline, after 8 and 16 sessions, at the end of therapy, after 25 ± 3 sessions, and at 6, 12 and 36 month follow-up. Underlying mechanisms of change are investigated. Statistical analyses will be conducted using the appropriate multilevel approaches, mainly two-level regression and growth analysis.

**Discussion:**

The results of this study will indicate whether the integration of emotion-focused elements into treatment as usual increases the effectiveness of Psychological Therapy. If advantages are found, which may be limited to particular variables or subgroups of patients, recommendations for a systematic integration, and caveats if also disadvantages are detected, can be formulated. On a more abstract level, a cognitive behavioral (represented by PT) and humanistic/experiential (represented by EFT) approach will be integrated. It must be emphasized that mimicking common practice in the development and continued education of psychotherapists, EFT is not integrated as a whole, but only elements of EFT that are considered particularly important, and can be trained in an 8-day training plus supervision of therapies.

**Trial registration:**

ClinicalTrials.gov, NCT02822443, 22 June 2016, retrospectively registered

## Background

Grawe formulated an approach designated General Psychotherapy [1–3] in which he postulated that first generation approaches, the original approaches to psychotherapy as developed by their founders, had to be overcome. In his opinion they neglect or even suppress and fight concepts and findings that are not in line with their original stance. Second generation approaches, in contrast, utilize all concepts and evidence relevant for a scope of applications. The domain for which it claims relevance may be limited, but all that is relevant to the claimed range of application should be integrated. As research is continually developing, *General Psychotherapy* stands for a continuous endeavor despite the end state never fully being reached. It is not just another approach to psychotherapy with a fixed set of concepts and interventions, but rather a model in continuous development. *Psychological therapy* (PT; [4, 5]) as practiced in Bern at the outpatient clinic of the Institute of Psychology and taught in the postgraduate training program as well as in many other German-speaking institutions, follows the idea of General Psychotherapy. It is mainly a cognitive behavioral approach that has its roots in humanistic and learning theories, but also relies on cognitive science, emotion and social psychology, neurobiology, and interpersonal and systemic approaches. Since its origins in the late 70’s, there has been an ongoing attempt to follow the principles of General Psychotherapy. This has led to an approach that could be described as integrative [6]. The integration, however, is not eclectic but guided by theoretical concepts such as general change factors [4]. These change factors include clarification, resource activation, problem activation, and problem mastery. Psychotherapeutic interventions can be related to these factors, which allows for the description of approaches to psychotherapy in terms of their typical profiles. Cognitive-behavioral therapy (CBT), for example, has an emphasis on mastery, and problem activation takes specific forms, such as behavioral exposure. Systemic approaches have a traditional strength in resource activation. Client centered therapy and psychodynamic approaches predominantly offer interventions fostering clarification, etc. A problem is that not all patients need the same profile in their therapy, and matching the patients’ needs with what a traditional approach has to offer is not an optimal solution: The same patient may need different approaches for different problems, there may be a change of needs over time, and not all relevant problems may be known in the beginning of a therapy. Therefore a psychotherapeutic approach should be adaptable to the patient needs and possibilities as reflected in a case formulation [7]. To reach this goal, it is desirable that for all change factors a sufficient range of interventions and concepts upon which they are based is available, and the use of each has been empirically studied.

In the past decades Emotion-Focused Therapy (EFT) has become increasingly popular, both in clinical practice and in research. EFT is an approach of humanistic, client-centered, and gestalt origin. Main proponents are Greenberg, Elliott, Paivio, Watson, Pascual-Leone, Goldman, and Pos (for an overview: [8]). EFT refers to common concepts of emotion psychology and other relevant domains of psychology and includes a number of concepts as well as interventions. EFT is a process-oriented approach that integrates an empathic relationship offer and process-directive interventions aiming to improve a patient’s ability to constructively deal with emotions [9]. According to the prescriptive concepts of EFT, various types of emotional experiencing/processing are distinguished, which require different interventions. Important distinctions are primary vs. secondary emotions (roughly: natural/spontaneous vs. transformed/distorted) and adaptive vs. maladaptive emotions (roughly: helpful vs. not helpful for satisfying one’s needs). It is assumed that a patient’s problems are often related to an inability to understand own emotions and thus an inability to derive appropriate responses. It can also be an inability to expose oneself to threatening or painful emotions, even though such exposure has a potential of fostering personal development. The overarching goal is to enable the patient to become asymptomatic and improve quality of life by transforming maladaptive emotions into adaptive emotions. The therapeutic procedure is led by “markers” (indicators for problems in emotional processing, but also for a patient’s readiness to work on emotional problems), which become visible/audible in the therapeutic process and indicate which therapeutic interventions are most promising under which circumstances. Within a relatively short time, EFT has acquired a sound scientific stance in several empirical studies [10]. It corresponds to APA (American Psychological Association) standards of empirically validated treatments for individual treatment of depression and for couples therapy, for which manuals have been developed [9, 11–13]. Moreover, there is evidence for positive effects on other disorders.

In practice, psychotherapists increasingly tailor their interventions to the characteristics of an individual patient and thereby use a number of methods not confined to a single therapy approach. Recent evidence shows that a big part, if not a majority of psychotherapists, adopt a rather integrative stance [14]. Trained in one approach, therapists seek complements in other approaches when they find conceptual and practical weaknesses of their initial approach. With experience, therapists acquire elements from other therapy schools and traditions and thus become more flexible in the treatment of their patients, conceptually and technically. Therapists tend to integrate therapeutic elements from a new approach into the old one, once they were found effective through empirical evidence. They rather integrate elements of a new approach into an old one than changing completely from the original approach to another [15]. At the level of training, a recent study conducted in the United States showed that one third of the accredited training programs in psychotherapy offer mandatory or optional training in five major psychotherapy theories (psychodynamic theory, humanistic theory, cognitive theory, behavioral theory, systems theory), 90% reported teaching psychotherapy integration in one or more courses [16]. The majority of trainees characterizes their therapeutic approach as “eclectic/integrative” [16], and in private practice, only two percent of therapists completely identify themselves with one single orientation [17]. A common type of integration has been named assimilative integration, that is, therapists are trained in a particular approach and take it as a point of departure for integrating other concepts and interventions that appear as particularly useful complements to the original one [15]. A recent expert panel on the future of psychotherapy in the United States of America (“Psychotherapy in 2022“) estimated a likely increase of integrative approaches [18].

Nevertheless, it is uncommon to study such integration, and research on its effects on process and outcome is rather rare [16]. Thus, more research on psychotherapy integration is needed, if psychotherapy research is to cover real practice in an endeavor to reduce the currently much bemoaned scientist-practitioner gap. The main aim of this study is to compare Psychological Therapy corresponding to the usual practice in Bern to Psychological Therapy with integrated EFT elements. A central characteristic of the presented project is its external validity being particularly evident in the elaboration of naturalistic conditions and treatment as usual (TAU) being part of both conditions (TAU + EFT and TAU). Twenty-three therapists per condition treat a total of 130 patients from the outpatient clinic of the University of Bern, suffering from depressive, anxiety and adjustment disorders. Therapists vary in their general therapy experience and extent of training. This will allow for evaluating the influence of these variables. To secure balance regarding the amount of training and supervision between the project conditions, TAU without EFT will be supplemented with additional units elaborating on some elements that are already part of PT.

The overarching question addressed is: What are the consequences of systematically integrating emotion-focused concepts and interventions into Psychological Therapy? This is seen as exemplary for major steps in therapy development in the sense of General Psychotherapy and follows suggestions by others [19]. The general research question can be subdivided into the following questions:

1. Is there a general superiority of TAU + EFT over TAU in the changes from pre to follow-up (with indicators such as stability of change, post-therapeutic gain, and reduction of relapses)?
2. Is there a superiority of TAU + EFT over TAU in variables indicating deeper levels of processing?
3. Are there negative side effects of the integration e.g. due to less attention and time dedicated to more traditional but useful elements and procedures?
4. Additional exploratory research questions include the examination of potential predictors, moderators and mediators of outcome (e.g. symptom severity, onset of primary disorder, previous psychotherapies, and process variables such as experiencing ratings).

Some questions are specific in terms of differential effects regarding TAU and TAU + EFT (e.g., level of experiential processing, emotion-regulation skills). The example of emotional processing (EP) is used to illustrate the kind of planned analyses. EP is assumed to be a trans-theoretical mechanism of change [20] and emotion-focused interventions are considered potent ways to facilitate emotional processing [12, 21]. Moreover, the level of EP has predicted psychotherapy outcome in previous research [22, 23]. Therefore, we hypothesize that patients in TAU + EFT will show higher levels of EP than patients in TAU, and the level of EP in both conditions will mediate the relationship between emotion-focused interventions and therapy outcome. Higher levels of EP will predict better outcomes at follow-up.

## Methods

### Participants

A total of 130 patients fulfilling the diagnostic criteria for a unipolar depressive (ICD, F32), anxiety (ICD, F40, F41) or adjustment disorder (ICD, F43.2) are being recruited, with 65 participants randomly assigned to the TAU + EFT condition and 65 to treatment as usual. Participants are recruited at the psychotherapy outpatient clinic of the University of Bern, once they have registered for therapy and meet the requirements for participation in the study. As both conditions can be offered as treatments with empirically supported effects, it is not expected that many patients will decline, although the standard of 25 ± 3 sessions may be an obstacle to some.

With an average of three therapies per therapist, 23 therapists are needed per condition. In support of external validity and generalizability of our findings, therapists of varying experience are included. The participation of five experienced therapists and 18 therapists in training per condition is planned. All therapists in this study have a master’s degree in psychology and therapists in training have been in postgraduate training at the University of Bern for at least 1 and a half years.

### Inclusion and exclusion criteria

One important goal of this project is to inform therapists about the effects of integrating emotion-focused elements in Psychological Therapy in a naturalistic and routine practice setting. To maximize external validity and generalizability to common therapeutic practice the patient sample should not be too homogeneous and the sample should be replicable. A good solution seems to focus on patients with unipolar depressive, anxiety and adjustments disorders as the most prevalent patient groups in psychotherapy outpatient settings [24], making about 50% of the patients in our outpatient clinic. Minimum age is 18. Exclusion criteria are active substance dependence for the previous 6 months, current suicidal risk or immediate threats of self-harm, or meeting criteria for organic mental disorders. In addition, we exclude individuals with health conditions that require medication potentially affecting their mood (e.g., steroids), and individuals receiving concurrent psychological treatments, including psychotherapy. Patients who have been under antidepressant medication at a stable dose for at least 1 month are allowed to participate. Comorbidity with disorders not on the exclusion list does not lead to exclusion as long as anxiety, depression, or adjustment problems are of primary concern.

### Sample size calculation

Based on a power analysis with G*Power [25] an optimal total sample size of 130 patients allows for the detection of a small effect (Cohen’s f = 0.10) for the interaction between time (pre, post, follow-up) and treatment condition (TAU + EFT vs. TAU) (repeated measures analyses of variance (ANOVA), within-between-interaction; *α* = 0.05; power = 0.80; number of groups = 2; number of measurements = 3; pre-post correlation of pre-post values: *r* = 0.6; non-sphericity correction = 1). Multilevel models allow for assessment-by-assessment approaches: Assuming 130 patients and three assessments per patient (pre, post, follow-up), the resulting N would be 390. Further assuming a 20% dropout rate at follow-up, this sample would be reduced to 312. This would enable the detection of small effects of 3.4% explained variance in a regression model (linear multiple regression: random model; H1: ρ2 > 0; α = 0.05; power = 0.80) with three predictors (treatment condition, time, and their interaction); and would still allow the identification of small effects of 5.1% explained variance in a model including additional covariates with a total of ten predictors [25].

### Study design and group allocation

This study is conducted as a randomized controlled trial with two active treatments: TAU + EFT and TAU. A 2 × 3 design is used with one between-subject factor (two treatment conditions) and one within-subject factor (time: pre, post, 12 month follow-up).

**Fig. 1:**
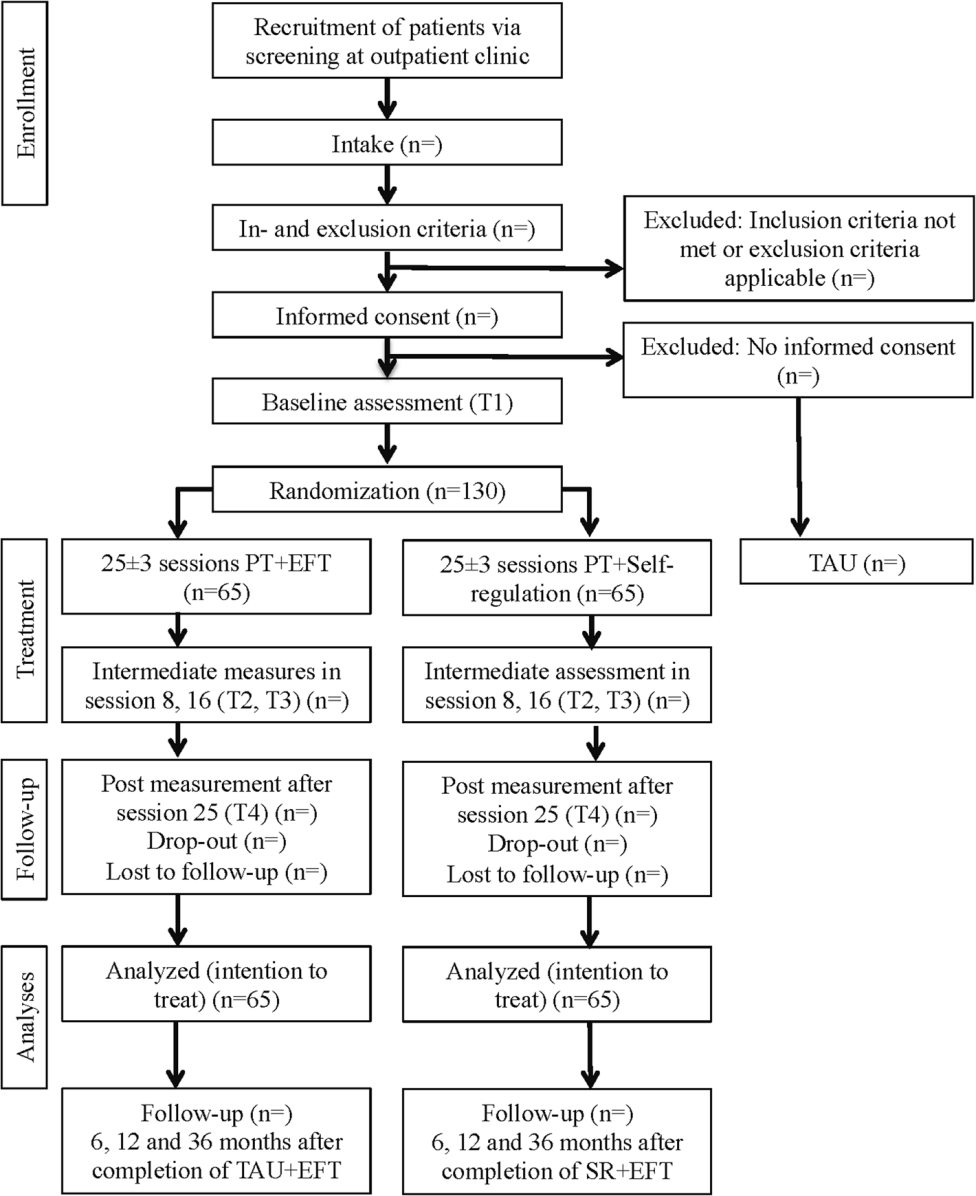
Participant recruitment and study flow chart

After completion of the baseline assessment and checking of the inclusion and exclusion criteria, a randomization procedure with equal allocation of patients to each treatment condition is used. To ensure a balanced distribution of diagnostic groups in the two treatment arms, a stratified randomization is applied. The allocation lists are created by an independent researcher with a computerized random number generator and are unknown to the investigators. The study design is shown in Fig. 1.

### Description of the interventions

The treatment conditions are Psychological Therapy as usual and Psychological Therapy with emotion-focused components. Each intervention consists of 25 ± 3 sessions of 50 min each. Twenty-five sessions is the official standard for short-term therapies in the German health-care system. To standardize the duration to some extent serves to facilitate the comparison of therapies in the planned process analyses.

*Case formulation and Plan Analysis:* Both treatments are based on Psychological Therapy [5], an integrative form of cognitive-behavioral therapy and are based on explicit individual case formulations. The case formulations include an analysis of the individual etiology for the development and maintenance of patient problems. A first overarching question is which factors lead to inconsistency (i.e., the tension resulting from discrepancies between needs and reality and from internal conflicts [4]). Inconsistency has been shown to be closely related to mental problems [26]. Second, patient strengths and resources are emphasized and used (e.g., abilities, preferences, favorable circumstances, etc.). The patient’s ability to secure and enhance consistency and to solve problems is conceptualized in terms of Plan Analysis [27]. The case formulation also includes an analysis of problems and potentials for the therapeutic relationship.

Psychological therapy further makes reference to general change factors, and an explicit prescriptive concept for fostering the therapeutic relationship (Motive-Oriented Therapy Relationship as derived by the therapist from Plan Analysis). The main focus of Plan Analysis [27] is the instrumentality of behavior and experience (what conscious or non-conscious purpose does an aspect of overt or covert behavior hypothetically serve?). From the patient’s verbal and nonverbal behavior, the therapist infers underlying Plans of which many are non-conscious. For a specific patient, the therapist defines and implements a customized therapeutic relationship offer based on an individual Plan Analysis. Whereas the Motive-Oriented Therapy Relationship is a prescriptive approach, it is neutral in terms of therapy orientations. Its essence is to recognize, support and foster a patient’s positive motives in an active way that is not contingent to the presenting problem behaviors. Whereas the therapeutic procedure is developed individually, it utilizes etiological models and therapeutic procedures as often described in manuals. Following the principles of General Psychotherapy, the choice of helpful concepts and interventions is generally free, but empirical evidence is a strong argument for the therapist to favor one over another. In principle, all change factors, clarification, resource activation, problem activation, and problem mastery [4], are utilized, and the whole range of broad-spectrum behavior therapy interventions may be implemented [28–30]. However, it has been found in the past that working with emotions (instances of the change factors problem activation and clarification) has normally less weight in comparison to more cognitive/rational forms of clarification, skill building, or behavioral exercises. As explained above, this is closely related to lesser familiarity with and a greater insecurity in the implementation of interventions focusing on emotions.

*Treatment as usual with emotion-focused components* [8] is based on Psychological Therapy, but emphasizes working with emotions, particularly the use of EFT models and techniques. This involves the practice of mainly four psychological skills: empathy, focusing, two-chair work and empty-chair work. These are conveyed in a special 8-day training and supported by supervisions (individually or in small groups of up to four supervisees) on average every 2 weeks, so that therapists feel comfortable using them. In addition every 3 months a supervision of the supervisors by expert EFT supervisors takes place. Manuals, which are relatively heuristic/flexible to allow for individualized procedures, accompany instructions for the interventions. All components of both interventions must be implemented according to the manual’s specification. For detailed information on the content of EFT-components see Table 1.

**Table 1:** Content of the emotion-focused components

EFT-Component	Content
Empathy	Empathy forms the basis of the therapeutic work in emotion-focused therapy as a technique and the fundament of the therapeutic relationship. Different forms of empathy play an important role in the shaping of the therapeutic relationship, affect regulation, deconstruction and the establishment of positive behavior towards the self.
Focusing	Focusing is a therapeutic technique to help expand the cognitive memories by the corresponding bodily reactions and thereby activate affective schemes usually arising in problematic situations. The goal is to look at current behavior in a larger context and recognize potential relationships to past experiences.
Two-chair work	The two-chair dialogue is used for confrontational processes e.g. self-evaluative splits, anxiety-splits and hopelessness splits where the patient operates alternating from both chairs. The main aim of two-chair work is an increase in self-compassion.
Empty-chair work	An indication for the empty-chair work is unfinished business with a significant other. The significant other can be imagined in the empty chair and contacted. The objective is a change in emotional schemes concerning the significant other.

*Treatment as usual:* In an add-on design, it would be problematic to give special training and attention to therapists only in one condition, because it would be hard to retrospectively single out factors such as higher expectancy, additional investment of time, allegiance, etc. To ensure that effects are specifically attributable to the add-on condition, it is important to balance out the conditions by making an equivalent addition also to the TAU condition, while keeping these additions within the concepts that characterize TAU. Thus in the TAU condition, self-regulatory processes as conceptualized by Carver & Scheier [31] and others receive particular attention as an equivalent addition. Self-regulatory processes are part of the consistency theory described by Grawe [4] and are conceptually part of Psychological Therapy as usual. It has been found though, that therapists seldom exploit the concrete possibilities of utilizing the self-regulation perspective in practice. Therefore, concrete self-regulation based interventions including psychoeducation on self-regulation models have been described and conveyed in the training. The self-regulation perspective does not come along with specific interventions. However, the self-regulation perspective determines the planning of interventions in this condition, the way therapists are conveyed to their patients, and the choice of an attention focus.

In addition, therapists in this condition are advised to use strategies emphasizing emotions not more than considered necessary based on the individual case conceptualization. The first category in Table 1, empathy, is considered to be part of TAU, although plausibly more typical and frequent in the TAU + EFT condition. Techniques most typical for EFT (categories 2–4 in Table 1) are proscribed although in the improbable case that a therapist thinks, that an intervention typical for EFT is absolutely required for a particular patient, he or she can argue in favor of such an intervention vis a vis the supervisor who can approve it, if convinced that no non-EFT procedure would lead to similar effects.

The amount of training and supervision is equivalent in both conditions. Besides the basic model of self-regulation by Carver & Scheier [31] other concepts are part of this active control condition, e.g. practicing an inner monologue for the planning and regulation of behavior [32] and clarification which factors lead to maladaptive self-organization, in particular ego depletion [33]. For a detailed description of the self-regulation components see Table 2.

**Table 2:** Content of the self-regulation components

SR-Component	Content
Explanation of the SR-model	Explanation and discussion of the basic model of self-regulation. Illustration of both, self-regulatory and self-organized processes. Responding to the different boxes in the model and development of possible therapeutic starting points.
Clarification, when the patient produces perceptions, instead of objective change	Identification of changes reducing discrepancies between desired and the perceived states in perception only, as opposed to more tangible, concrete changes.
Deliberate reflection of goals and values	Goals, values, needs and standards are brought to mind and reflected. Finding out possible meanings for the activity of the comparator (which compares perceived to desired states).
Tracing the development of ideals and norms from personal history	Clarification of the origin of goals, values, needs and standards from the biography of the patient.
Attention-regulation	Training of conscious adaptation of the allocation of attention to the requirements and the switching between different modes of perception (deliberate/conscious vs. implicit/self-organized). Focusing attention on self-organized patterns of attention.
Work on self-instruction	Practicing self-control by the concretization of long-term consequences, to strengthen them over short-term consequences.
Regulation of behavior	Learning to monitor and control own behavior in terms of dual-process models (deliberate vs. self-organized regulation).
Regulation of the body	Relaxation exercises and techniques to reduce tension and agitation.
Emotion-regulation	Training of skills in emotion regulation as part of self-regulation.

### Measurements

For an overview of assessments at baseline, intermediate measurements (8 weeks, 16 weeks), post-treatment after 25-weeks, as well as 6, 12 and 36 month follow-up see Table 3.

**Table 3:** Measurements and time of assessment

Instrument	Abbr.	Aim	Time of assessment
Clinician administered			
Structured Clinical Interview for DSM IV	SCID	DSM-IV Axis I/II disorders	pre, post
Hamilton Depression Rating Scale	HDRS	severity of depressive symptoms	pre, post
Goal Attainment Scaling	GAS	individual goals	pre, intermediate, post
Self-report ratings			
A. Symptom severity			
Brief Symptom Inventory	BSI	symptom impairment	pre, intermediate, post, follow-up
Beck Depression Inventory	BDI-II	severity of depressive symptoms	pre, intermediate, post, follow-up
Beck Anxiety Inventory	BAI	severity of anxiety symptoms	pre, intermediate, post, follow-up
B. Wellbeing			
World Health Organization 5	WHO-5	psychological wellbeing	pre, intermediate, post, follow-up
Short Form 12 of the Health Survey	SF-12	health-related quality of life	pre, intermediate, post, follow-up
C. Coping/Emotion regulation			
Self-assessment of Emotional Competences	SEK-27	dealing with negative emotions	pre, post
D. Interpersonal problems			
Inventory of Interpersonal Problems	IIP-32	interpersonal problems	pre, intermediate, post, follow-up
E. Motives/Incongruence			
Inventory of Approach and Avoidance Motives	FAMOS	motivational goals and schemes	pre, post
Incongruence Questionnaire	INK	incongruence	pre, intermediate, post, follow-up
F. Process measures			
Bern Post-Session Report Patient Version	BPSR-P	treatment process	after every therapy session
Bern Post-Session Report Therapist Version	BPSR-T	treatment process	after every therapy session
Symptom Checklist	SCL-9	psychological distress	after every therapy session
Classification of Affective Meaning States	CAMS	emotional processing	rating of therapy session
Experiencing Scale	EXP	experiencing	rating of therapy sessions

*Abbr.* Abbreviation

### Primary outcome measures

Measures of psychopathology, symptoms of depression and symptoms of anxiety are used as a composite primary outcome measure [34]. This composite measure consists of the Brief Symptom Inventory [35], the Beck Depression Inventory II [36] and the Beck Anxiety Inventory [37].

#### Brief symptom inventory

The Brief Symptom Inventory (BSI; [35]) is a self-report measure consisting of 53 items and detecting the subjective impairment by a range of psychological symptoms during the last seven days. The BSI offers information about the psychological burden with regard to nine subscales: somatization, obsessive-compulsive, interpersonal sensitivity, depression, anxiety, hostility, phobic anxiety, paranoid ideation, psychoticism. As an economic screening instrument with robust psychometric properties, this inventory is commonly administered to detect pre-post changes [35].

#### Beck depression inventory II

The revised version of the Beck Depression Inventory (BDI-II; [36]) is a self-assessment tool consisting of 21 items to determine depressive symptoms during the past 2 weeks. The BDI-II is not only an indicator of the severity of depressive symptoms in accordance with DSM-IV but also one of the most widely used self-report measures for depression in clinical practice and research [38]. It has shown robust psychometric properties [36].

#### Beck anxiety inventory

The Beck Anxiety Inventory (BAI; [37]) is a self-report questionnaire to detect the severity of anxiety symptoms. The BAI consists of 21 descriptive statements with regard to symptom severity during the last 7 days. 13 of 21 items detect physiological symptoms, five items measure cognitive aspects of anxiety and three items refer to both, somatic and cognitive symptoms. The BAI can be cited as a reliable and valid questionnaire [37].

### Secondary outcome measures

#### World Health Organization

The WHO-5 [39] is a short questionnaire measuring subjective psychological wellbeing over the past 2 weeks using five items. A low value indicates low wellbeing and quality of life and a high value is associated with wellbeing and high quality of living. The WHO-5 has shown to be a sensitive and specific screening instrument for depression [40]. The clinimetric validity, the responsiveness and sensitivity were evaluated. The WHO-5 performed well with regard to all these aspects [40].

#### Short Form of the Health Survey

Health-related quality of life is measured with the Short Form of the Health Survey (SF-12; [41]). Its two subscales measure physical and mental aspects of health-related quality of life. It captures general health as well as pain, disabilities in daily life and mental problems. The SF-12 asks for the presence and severity of 12 items over the course of the last 4 weeks. The re-test reliability is good and roughly equivalent to the long form [42].

#### Emotional competence

Emotional Competence is measured by the SEK-27 [43]. The emotional competence is recorded both, in general (trait) as well as with respect to the last week (prolonged state). The questionnaire consists of 27 items that are resumed to nine subscales: attention, clarity, body perception, understanding, acceptance, resilience, self-support, willingness to confront and regulation. The total value generally corresponds to the constructive handling of negative emotions. The SEK-27 is a reliable, valid and sensitive self-assessment measure for the constructive dealing with negative emotions [43].

#### Inventory of interpersonal problems

The Inventory of Interpersonal Problems (IIP-32; [44]) is a questionnaire for the self-assessment of interpersonal problems. With the help of this instrument patients can describe how much they suffer from specific difficulties in dealing with other people. The IIP-32 consists of 32 items and the eight scales correspond to the octants of the Interpersonal Circle [45]: too autocratic/dominant, too expressive/intrusive, too caring/friendly, too exploitable/resilient, too insecure/obsequious, too introverted/socially avoidant, too repellent/cold, too quarrelsome/competitive. In addition, a total value is formed which characterizes the degree of interpersonal problems. The IIP-32 has shown adequate psychometric properties [46].

#### Inventory of approach and avoidance motives

The Inventory of Approach and Avoidance Motives (IAAM/German: FAMOS; [47]) assesses motivational goals of psychotherapy patients. The FAMOS consists of 94 items, which are rated in terms of their importance. The motivational goals are differentiated into approach-goals (14 scales; intimacy, socializing, helping others, recognition, impressing, autonomy, performance, control, education, faith, variety, self-confidence, self-rewarding) and avoidance-goals (nine scales; loneliness, contempt, humiliation, criticism, dependence, tension with others, being vulnerable, helplessness, failure). The FAMOS is both, a diagnostic tool in the context of treatment planning as well as a measure of change throughout psychotherapy and has shown good psychometric properties [47].

#### Incongruence questionnaire short version

The Incongruence Questionnaire Short Version (K-INK; [48]) is a procedure for the determination of incongruities between the perceived reality and the motivational goals of psychotherapy patients. The K-INK is based on the Inventory of Approach and Avoidance Motives [47] and the consistency theory by Grawe [5]. The short version of the INK includes the 23 items of the long version with the highest item-total correlation with each of the 23 INK-scales, whereby 14 target the approach-goals and nine items target the avoidance-goals. The INK is the second questionnaire to attempt the building of a test-theoretical basis for Grawes psychotherapy research approach and has shown good psychometric properties [48].

### Clinician administered measures

#### Structured clinical interview for DSM-IV

The patients’ diagnostic status at baseline will be assessed with an interview of about one and a half hours conducted by trained raters (therapists in training) using the Structured Clinical Interview for DSM-IV (SCID; [49]).

#### Hamilton depression rating scale

The Hamilton Depression Rating Scale (HAMD; [50]) is administered together with the SCID. It is a well-established clinician-rated assessment of depressive symptom severity and encompasses psychological and somatic symptoms. The clinician rates the severity of these symptoms based on patient reports and his or her own observation.

#### Goal attainment scaling

The Goal Attainment Scaling (GAS; [51]) is a tool for the definition of individual goals and the evaluation of goal attainment in psychotherapy. The patient can indicate to what extend he/she was able reach the individual goals that were formulated at the beginning of psychotherapy on a 7-point scale from -2 to 4. Point 0 describes the current state of the problem, point +4 describes the desirable state and -2 the state if the problem deteriorated. The GAS interview is conducted with the patient by trained Master students.

### Process measures

#### Bern post-session report

The Bern Post-Session Report (Patient and Therapist Version; BPSR-P/BPSR-T; [52]) is an instrument for the assessment of treatment processes and a regular quality-monitoring tool, completed at the end of each therapy session. The patient version consists of 32 bipolar items which are rated on a scale ranging from -3 = not at all to +3 = yes exactly. The subscales include resource activation, positive bonding experiences, positive therapeutic relationship, problem mastery, positive problem solving experience, positive clarification experiences and treatment progress.

The therapist version assesses the treatment processes from the therapists’ perspective and consists of 27 bipolar items, which are also rated at the end of each therapy session. The subscales include resource activation, therapeutic relationship, openness and engagement, willingness to work hard, problem mastery, problem solving, motivational clarification, treatment progress, interactional perspective and interactional difficult. Further, new items concerning the study-specific interventions were added to the Bern Post-Session Report Therapist Version (see Table 4).

**Table 4:** Checklist of the study-specific interventions implemented in the therapy session

Today I conducted emotion-focused intervention(s)	
If so, which emotion-focused interventions (empathic exploration, empathic validation, engendering of a medium degree of emotional activation, focusing, allowing and expressing emotions, biographical work, systematic evocative deduction, two-chair dialogue, empty-chair dialogue, other Emotion-focused intervention)?	
Today I conducted intervention(s) to improve self-regulation (SR)	
If so, which interventions fostering self-regulation (explanation of the SR-model, clarification, deliberate reflection of goals and values, derivation of ideals and norms from personal history, attention-regulation, work on self-instruction, regulation of behavior, regulation of the body, emotion-regulation, other self-regulatory interventions)?	
Has it been difficult to integrate emotion-focused components into today’s therapy?	
If so, which difficulties occurred?	
Has it been difficult to integrate self-regulation into today’s therapy?	
If so, which difficulties occurred?	
Did you have reasons to not realize any study-specific interventions?	
If so, which reasons would that be?	

#### Symptom check list

The Symptom Checklist - 9 (SCL-K-9; [53]) is a short form of the revised Symptom Checklist (SCL-90), which in turn is a previous version of the Brief Symptom Inventory. The results of the SCL-K-9 on session-level thus correspond to the results of the BSI total score (General Symptom Index; GSI) as a primary outcome measure (measured at pre, post and follow-up). The SCL-K-9 assesses the construct of psychological distress through symptom severity. The SCL-K-9 is composed of nine items corresponding to the nine scales of BSI and SCL (see above). It is a reliable and valid instrument that is used in clinical diagnostic and in practice as a measure of quality assurance [54].

#### Classification of affective meaning states

The Classification of Affective Meaning States (CAMS; [55]) is a process rating system for the systematic identification, observation and measurement of distinct emotional states in psychotherapy sessions. This observer-based rating system was developed based on emotion-focused theory [9]. The CAMS assesses ten affective meaning states that can be ordered on nine different levels of emotional transformation referring to a sequential model of emotional processing [21]. In several studies an excellent inter-rater reliability was reported [56].

#### Experiencing scale

The Experiencing Scale (EXP; [57]) is a rating scale assessing the degree to which clients orient to, symbolize, and use internally felt experiences as a source of information when solving their problems. Raters use verbal communication, including features of content, expression, grammatical selection and paralinguistic to code segments of therapy. Ratings on the lower scale levels represent clients’ attempts to identify and symbolize their internal experience. Higher scale levels by contrast reflect the clients’ efforts to use an experientially- oriented understanding for problem solving. The Experiencing Scale stands among the most studied and validated observational measures in psychotherapy research [57].

Depth of change will be measured by the observer-rated CAMS and EXP as well as by patient and therapist rated process questionnaires (e.g. problem actuation, clarification, emotional processing, and experiencing).

### Procedure

**Fig. 2:**
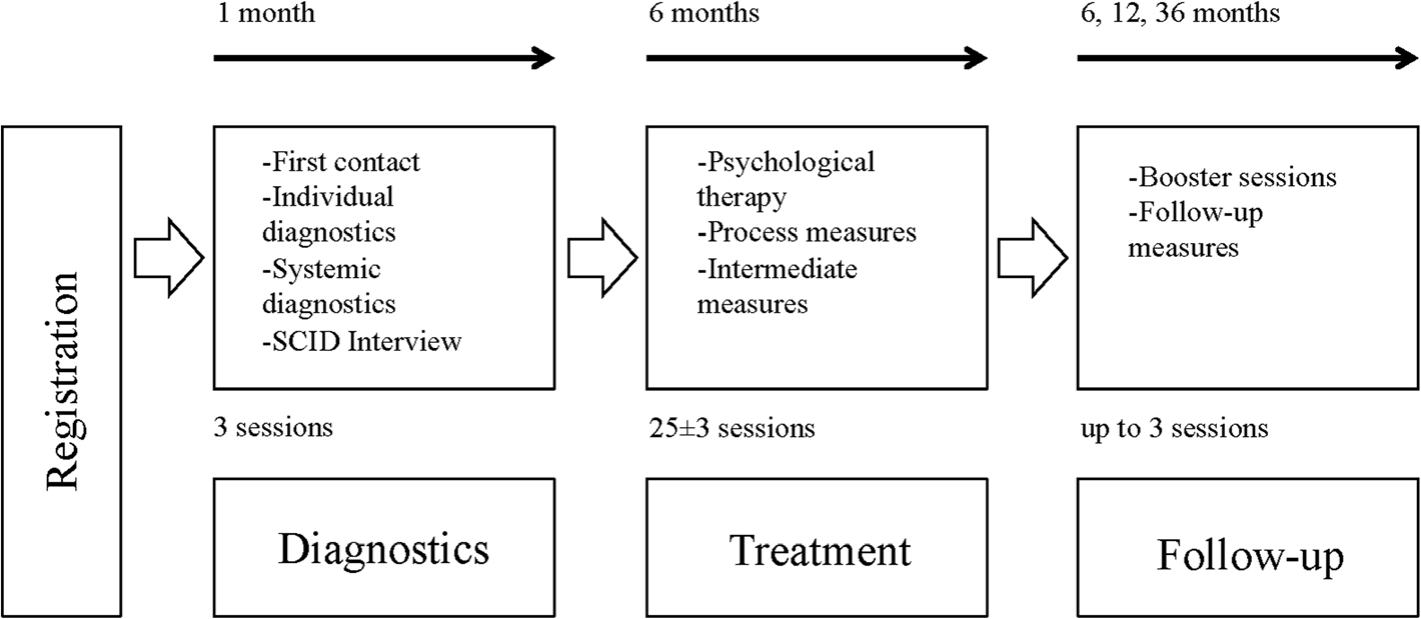
Procedure of the study

Patients are randomly assigned to the TAU + EFT or TAU condition. The patients receive 25 ± 3 sessions of weekly Psychological Therapy with or without integration of EFT elements. Both groups are assessed at baseline (t0), immediately after completing therapy (t3, 25 sessions), for intermediate measurements (t1, 8 sessions; t2, 16 sessions) and at 6, 12 and 36 month follow-up (t4, t5, t6) with an elaborated measuring battery (see Table 2). Additionally, participants and therapists complete self-report measures after every session for the detection of the treatment process and symptom severity. All data will be saved in an anonymous way only identified by a code, which is not related to the participant’s identity. Servers are protected by high-end firewall systems. Only the researchers directly involved in the study have access to the data. The procedure is shown in Fig. 2.

### Analysis

Baseline descriptive statistics will be generated for all randomized patients and compared between the two study arms with ANOVA (for continuous variables) and χ2 statistics (for categorical variables). Missing values will be substituted with the procedure of multiple imputation. The research questions will be examined with the appropriate multilevel approaches, mainly two-level regression and growth analyses. These approaches take into account non-independence of observations in repeated measures outcome and the different number of sessions attended by the patients. Furthermore, we intent to test potential variability within therapists based on a longitudinal three-level model. The primary outcome analysis will be a modified intention-to-treat analysis that includes all patients who were randomized and attended at least one therapy session. These analyses will compare treatment differences in continuous outcome variables over time for TAU + EFT and TAU. Separate multilevel analyses will be run for the primary and each of the secondary outcome variables across three time points (pretreatment, post- treatment, 12-month follow-up). We expect primary and secondary outcome measures to be highly inter-correlated loading on one outcome factor [34]. For the purposes of the present study, a standardized composite measure taking primary and secondary symptom-related, self-report measures into account will be reported. Models will be run assuming random intercepts and slopes. For the main research questions, level-one models of individual change over time and level 2 models for the between-subjects factors are conducted. Each analysis will examine the overall effect of change over time (time), the difference between TAU + EFT and TAU, and the differences in changes over time by condition as a cross level interaction. To assess maintenance of gains, the multilevel regression analyses will be repeated with just the post-treatment and follow-up time points. A secondary series of analyses will include only those patients who completed the originally allocated treatment. Mechanisms of change will be examined as mediation effects in multilevel regression and structural equation models. Moderator effects will be analyzed as cross level interactions. Therapist effects will be investigated in three-level models. Multiple regression models will be used to predict residual change in the composite score between post and follow-up, by the level of structural change at post-treatment.

## Discussion

In this randomized controlled trial, the effectiveness of treatment as usual with integration of emotion-focused components (TAU + EFT) and TAU is compared. The originality of this project lies in the examination of the consequences of integrating interventions of another promising evidence-based approach (EFT) into treatment as usual in a way that is directly mirroring common integrative practice. The use of an elaborated and intensively used psychotherapeutic model (TAU) speaks for a general effectiveness of both conditions. Emotion-Focused Therapy has acquired empirical validation for the treatment of depression, trauma and abuse [12]. Clinically significant improvements with substantial effect sizes for both treatments in primary and secondary outcome measures are thus expected.

Other projects dealing with the integration of EFT elements [12, 22, 23, 58] did not report great differences in effectiveness. Newman and colleagues for example [58] compared an integrative psychotherapy of generalized anxiety disorder that added EFT and interpersonal elements to a standardized CBT treatment with a treatment that added supportive listening to the same CBT component. The integrative therapy was equally effective post treatment and 2 years later, so that the authors concluded that the augmentation of CBT with emotion-focused and interpersonal techniques might not lead to better outcomes for generalized anxiety disorder patients. Similar results were found in an RCT on the treatment of patients with depression by Grosse Holtforth et al. [22], comparing Exposure-Based Cognitive Therapy (EBCT) with CBT. Component studies, which look at the effects of either adding particular techniques to a form of therapy (additive design) or taking them away (dismantling studies) rarely find that the presence or absence of specific techniques makes much difference to the overall outcomes [57]. In the history of psychotherapy, there are many examples of interventions that were less effective than expected, showed negative side effects, and worked in a different way than was believed [59, 60].

Grawe criticized what he called “the myth of an outcome equivalence, an artifact created by research design” [61]. There have in fact been some deficiencies in studies on comparative therapies that exacerbate the finding of specific change factors, e.g. the uniformity myth, small sample sizes, insufficient control of group assignments, disregard of competences and experiences of the therapists, inconsistent assessments of therapy success, lack of recording complementary interventions, differences in frequencies and durations of therapies, exclusion of drop-outs and missing of follow-up measures [62]. One point of criticism viewed alone results in considerable limitations on the validity of studies. In the summation of individual points of criticism doubt should arise on the general meaningfulness of the results.

From a General Psychotherapy perspective, newness is always part of a continuous development, of which the integration of a complementary concept with the potential of enriching an existing one can be an important step. This is a methodologically challenging endeavor, and this is a major reason why a relevant part of contemporary psychotherapy practice is not empirically examined. The application of pure approaches can be studied more easily, and consequently more evidence exists relating to such applications. The problem is that in clinical reality, a majority of practitioners do not apply pure approaches, partly because they question their relevance for routine practice. The endeavor of studying an integrative procedure corresponding to widespread practice requires not only an appropriate design but also a group of researchers possessing first-hand clinical knowledge in each of the conditions under investigation. Another requirement is motivated therapists being trained in practicing integrative therapy and at the same time, being able and willing to skillfully implement the procedures defined by the experimental the conditions. Finally, to render such a study realistic, an institution is highly desirable in which a practice similar to the one required by the study design is already well-established routine.

An obvious question is, of course, what will be different in the current study? This project is characterized by highly naturalistic conditions and thus it can be considered a major step towards closing the science-practitioner gap with respect to psychotherapy integration. On average, therapists will be more experienced and better trained than in previous studies. Certified EFT trainers including Dr. Greenberg have conducted the training. The supervisors have completed an advanced EFT training. Fostering external validity, therapies will be conducted in a regular treatment setting, and the inclusion of EFT will correspond more to regular practice. This will make a competent implementation easier and the procedures will be better integrated in an overarching model. It should be emphasized again that this is not a comparison of complete and pure EFT (which would require more extensive training) with treatment as usual. The spectrum of diagnoses will be larger, therapies will be somewhat longer, and the change processes will be studied extensively. Furthermore, the proposed study uses multilevel models to analyze treatment outcomes, hypothesized moderators and mediators, as well as therapist effects. While this approach is not yet common practice in randomized controlled trials (RCTs), it is very flexible, and exposes new perspectives on predictors of change at the within-person and the between-person level in the psychotherapeutic process.

A methodologically fundamental question is how therapist variance shall be controlled. It may seem like an ideal solution to let the same therapists conduct therapies in both conditions, and some studies actually use this strategy [22, 58]. However, having the same therapists in both conditions does not necessarily ensure that their preferences, belief in the methods, fit of the personal profile with the method, competencies etc. are equal between the two conditions, but may vary between the two conditions within one and the same therapist. In addition, it has been argued plausibly that there may be considerable carry-over effects when using therapists in more than one condition [63]. Whereas both options seem viable, we decided in this trial to control at the level of relevant psychological variables. Therapist variables (e.g., therapist experience in the respective condition) will be assessed, and their impact on differential change in the outcome variables will be investigated and taken into account in the interpretation of potential differences between the groups. We will also be able to test for differential effects, e.g. whether good effects depend on therapist experience in one but not the other condition. Also higher order interactions can be studied, e.g. whether the readiness of a particular kind of patient to engage in particular interventions depends on the perceived therapist competence, etc.

To conclude, an essential contribution of this study will be to better understand how an existing and well-elaborated psychotherapy approach may be further enriched by the integration of new elements. In addition to studying the effectiveness of the two treatment protocols, the current study examines unique and joint factors which moderate and mediate treatment effects in TAU + EFT and TAU. Furthermore, predictor variables are not only assessed before and after treatment but also over the course of treatment through weekly process measures. This provides the opportunity to measure temporal precedence and to make inferences about causality. We hope that insights into which treatment works best for whom and how, will help improve the care for patients with depressive, anxiety and adjustment disorders. Furthermore, the results of this study promise to indicate whether an 8-day EFT-training plus supervision can enhance the effectiveness of treatment as usual. Such an add-on format, if shown effective, would represent a “light” alternative to the full EFT-training, which may be more realistic and attractive for many therapists and would therefore contribute to a deserved larger implementation of EFT concepts and interventions into psychotherapy. The procedures and training could also be modified to treat other conditions as well.

### Trial status

Trial start date: 2 February 2016.

Currently recruiting (*N* = 28, 13 November 2016).

## Abbreviations

ANOVA: Analysis of variance
BAI: Beck anxiety inventory
BDI: Beck depression inventory
BPSR-P/BPSR-T: Bern post-session report patient version/Therapist version
BSI: Brief symptom inventory
CAMS: Classification of Affective Meaning States
CBT: Cognitive-behavioral therapy
EFT: Emotion-focused therapy
EXP: Experiencing scale
FAMOS: Inventory of approach and avoidance motives
GAS: Goal attainment scaling
HDRS: Hamilton depression rating scale
ICD: International classification for disease
IIP: Inventory of interpersonal problems
INK: Incongruence questionnaire
PT: Psychological therapy
SCID: Structured clinical interview for DSM-IV
SCL-9: Symptom checklist
SEK-27: Self-assessment of emotional competences
SF-12: Short Form 12 of the Health Survey
SR: self-regulation
TAU: treatment as usual
WHO-5: World Health Organization 5
